# Medicinal Plants with Prospective Benefits in the Management of Peptic Ulcer Diseases in Ghana

**DOI:** 10.1155/2021/5574041

**Published:** 2021-05-05

**Authors:** Mavis Boakye-Yiadom, Doris Kumadoh, Emmanuel Adase, Eric Woode

**Affiliations:** ^1^Clinical Research Department, Centre for Plant Medicine Research, Mampong-Akuapem, Ghana; ^2^Department of Pharmacology, Faculty of Pharmacy & Pharmaceutical Sciences, College of Health Sciences, Kwame Nkrumah University of Science and Technology, Kumasi, Ghana; ^3^Department of Pharmaceutics, Centre for Plant Medicine Research, Mampong-Akuapem, Ghana; ^4^Department of Production, Centre for Plant Medicine Research, Mampong-Akuapem, Ghana; ^5^Department of Pharmacology and Toxicology, School of Pharmacy, University of Health and Allied Sciences, Private Mail Bag, Volta Region, Ghana

## Abstract

**Background:**

The growth or multiplication of harmful microorganisms in addition to harmful human activities has led to many disorders in humans. Consequently, there is a search for medications to treat these disorders. Interestingly, medicines of plant origin are known to be among the most attractive sources of new drugs and have shown promising results in the treatment of various diseases including peptic ulcers. This review, therefore, is aimed at obtaining knowledge on some Ghanaian ethnomedicinal plants used to treat peptic ulcers, their folkloric uses, their phytochemicals, and their antiulcer and related pharmacological activities as well as finding areas for prospective studies.

**Methods:**

Published peer-reviewed articles on ethnomedicinal plants used for the management of peptic ulcers in Ghana from 1967 to 2020 were sourced and used for the study.

**Results:**

In this review, 13 plants were identified which belong to 10 different families including Sapindaceae, Apocynaceae, and Bignoniaceae. The parts most often used for most preparations were the leaves (53%), followed by stem bark and roots (both having the same percentage of use of 17.6%), the whole plant (5.9%), and the rhizomes (5.9%). *Azadirachta indica* was the only plant that had undergone some patient studies in addition to animal studies*. Conclusion*. A discussion of various antiulcer activity studies using ulcer models carried out on selected medicinal plants used for the management of peptic ulcer disease in addition to brief information on their folkloric uses and their phytochemical and other pharmacological properties is presented. These medicinal plants may be used in developing herbal products for the management of peptic ulcer disease.

## 1. Introduction

Peptic ulcer remains one of the gastrointestinal disorders that has affected many people worldwide over the centuries with over 5-10% of the world population generally affected. This condition occurs when the endothelial lining of the oesophagus, stomach, and/or duodenum is damaged. Also, insufficient gastric mucosal resistance may lead to peptic ulcers [1]. Some factors that cause peptic ulcers include *H. pylori* infection, alcohol consumption, tobacco smoking, psychological stress, some hereditary conditions, and drug abuse mostly with nonsteroidal anti-inflammatory drugs (NSAIDs) such as aspirin and ibuprofen [2]. Several orthodox drugs have been manufactured to combat this disorder. Most of these drugs are costly and have intolerable side effects when taken. Some orthodox drugs employed in the treatment of this disorder include histamine (H_2_) receptor antagonists, proton pump inhibitors, cytoprotectants, antacids, and prostaglandin analogues [3, 4].

Herbal medicines have been used in treating various diseases throughout the millennia before the discovery of most conventional medicines. Due to this, herbal medicines have been perceived to have healthier cultural acceptability and less toxic and decreased antagonistic effects [4]. Several prospective medicinal plants for peptic ulcer treatment have been studied and reported in literature globally. Lakshmi et al. reported on some medicinal plants including *Allophylus serratus* Kurz, *Cissus quadrangularis*, *Ocimum sanctum* Linn, *Mangifera indica* L., *Zingiber officinale* Roscoe, *Butea frondosa* Roxb. L., *Glycyrrhiza glabra* L., *Solanum nigrum* L., and *Terminalia chebula* Retz [5]. Also, medicinal plants such as *Cynodon dactylon*, *Azadirachta indica*, *Glycyrrhiza glabra*, *Swietenia mahagoni*, *Bauhinia purpurea* L., *Ficus religiosa*, *Melastoma malabathricum*, *Ocimum sanctum*, *Spondias mombin* L., *Eruca sativa*, and *Osyris quadripartite* have been reported as possessing some form of antiulcer activity upon investigation in antiulcer testing models [6–13]. A triple herbal therapy comprised of *Enterica*, *Dyspepsia* decoctions, and *Natural Pain Killer* (NPK 500) capsules manufactured by the Centre for Plant Medicine Research (CPMR) is currently used in the management of peptic ulcer disease at the CPMR out-patient clinic in Ghana. These three products contain medicinal plants such as *Carapa procera*, *Trichilia monadelpha*, *Persea americana*, *Trema orientalis*, *Momordica charantia*, *Vernonia amygdalina*, *Cassia siebieriana*, *Citrus aurantifolia*, *Bidens pilosa*, *Morinda lucida*, *Maytenus senegalensis*, *Psidium guajava*, *Cnestis ferruguinea*, *Spondias mombin*, and *Latana camara.* In this review, efforts have been made to present thirteen (13) prospective Ghanaian medicinal plants with their antiulcer activities. These medicinal plants are *Chromolaena odorata*, *Blighia sapida*, *Cyperus rotundus*, *Magnifera indica*, *Azadirachta indica*, *Alchornea cordifolia*, *Calotropis procera*, *Hoslundia opposita*, *Kigelia africana*, *Spathodea campanulata*, *Strophanthus hispidus*, *Zingiber officinale*, and *Paullinia pinnata.* These medicinal plants were selected due to their folkloric use in the treatment of peptic ulcers and availability in Ghana.

## 2. Literature Review Approach and Method Adopted

The information on ethnomedicinal plants used for the management of peptic ulcers in Ghana was obtained from published peer-reviewed articles on the plants. Electronic databases accessed included Google Scholar, PubMed, Web of Science, and Scopus. The major search terms used were “Plant names AND peptic ulcer,” “Plant names AND *Helicobacter pylori*,” “Plant names AND gastric ulcer,” “Plant names AND anti peptic ulcer,” and “Plant names and duodenal ulcer.” All the articles obtained were reviewed to get the needed information on ethnomedicinal plants used in Ghana for the management of peptic ulcers. A review was conducted on all of ethnomedicinal plants concerning their pharmacological activities (antiulcer activities) and active compounds that have been used in the management of peptic ulcers. Attempts at retrieving available preclinical and clinical works done on each plant were also done. The *in vivo* or *in vitro* methods employed by the researchers on each plant in their studies for peptic ulcer and the mechanism of action of each plant were assessed from the electronic data obtained. All the retrieved articles used were acknowledged and other evidence significant to each plant was provided such as type of plant extract, plant part used, scientific names, and antiulcer models.

## 3. Results

### 3.1. *Chromolaena odorata* (L.) R.M. King & H. Rob

*Chromolaena odorata* (*C. odorata*) from the Asteraceae family is a seasonal perennial flowering shrub that grows up to 2.5 m tall. The plant has a soft stem with hairy leaves which give an aromatic odour when prepared in a decoction form. *C. odorata* is mostly dominant in the tropical and subtropical areas and is domesticated in some parts of Asia, West Africa, and parts of Australia [14]. Local names for *C. odorata* include Siam weed, baby tea, devil weed, and paraffin bush [15]. *C. odorata* is traditionally used to treat diabetes, wounds, malaria, diarrhoea, dysentery, skin diseases, and toothache [16–18]. Several reviews have also shown that the plant has analgesic, antibacterial, antioxidative, anti-inflammatory, antifungal, and antidiabetic activities [19–24]. Aqueous and methanol extracts of the leaf have been investigated and found to have phytochemicals such as flavonoids, tannins, steroids, terpenoids, and cardiac glycosides. Alkaloids have also been seen in the methanolic extract [24–27].

The extract from the fresh leaves has been used for the treatment of various ulcers [28]. An evaluation of the cytoprotective effects of the ethanolic and aqueous extracts of *C. odorata* in combination with honey using absolute ethanol plus hydrochloric acid (HCl) for the induction of gastric wounds in Sprague-Dawley rats has been conducted. Results obtained indicated that *C. odorata* and honey may be useful in treating induced gastric membrane injury [29]. This could be attributed to the dominant anti-inflammatory and antioxidant properties of *C. odorata* [20, 22]. A study involving an investigation of the antiulcer effect of a combination of *Chromolaena odorata* and *Ocimum gratissimum* leaf extracts was reported to show a marked positive effect in the treatment of duodenal ulcer using the ethanol-induced ulcer model in adult albino Wistar rats. Pretreatment with the combined extracts was seen to minimise the formation of duodenal lesions in a dose-dependent fashion [30]. Paul et al. have reported on the antiulcer activities of *C. odorata* where stomach ulcer induced in rats using absolute ethanol was treated with aqueous extract from *C. odorata* [31]. A major reduction in stomach lesions using the ulcer index and histopathological data was observed in the treatment rats' group compared with the disease control group. The antiulcer action may be due to phytochemical constituents such as polyphenolic compounds and flavonoids contained in *C. odorata* [31]. Although the antiulcer activity of *C. odorata* has been confirmed, a report from a toxicity study conducted by Anyanwu et al. using the ethanolic extract of *C. odorata* at doses of 50 mg/kg, 100 mg/kg, and 250 mg/kg in albino rats indicated an increase in urea and creatinine levels in a dose-dependent manner coupled with the permeation of inflammatory cells and epithelial erosion in histological sections. They, therefore, concluded that uncontrolled use of the extract of this plant may have negative effects on the function of the kidney and the histology of the gut [32]. Doses for formulations must therefore be carefully selected with accompanying toxicological evaluations of finished products containing *C. odorata* extracts.

### 3.2. *Alchornea cordifolia* (Schumach. & Thonn.) Müll. Arg.

*Alchornea cordifolia* (*A. cordifolia*) from the family Euphorbiaceae is found widely in the African continent. It is a small perennial plant that grows up to 8 metres tall in marshy areas [33]. The plant is commonly known as the Christmas bush and propagated through sexual and asexual means. It is mostly used for the treatment and management of several diseases in Ghana. The stem and leaves are used as a folk medicine for the treatment of arthritis and muscle pains, venereal diseases, acute and inflammatory chronic conditions, cancer, ulcers, rheumatism, sores of canker, colds, miscarriage, urogenital infections, and leprosy and as an antidote for snakebite, bronchitis, cough, angina, headache, diarrhea, dysentery, fever, malaria, ringworm, and piles [34–38].

Pharmacological activities have been reported on *A. cordifolia* including analgesic, anxiolytic, anti-inflammatory, antibacterial, antidiabetic, antimicrobial, antimalarial, antiaflatoxicosis, and antioxidant activities [39–42]. Phytochemical analysis of the plant indicated the existence of tannins, terpenoids, glycosides, alkaloids, saponins, and flavonoids [43]. The methanolic extract of *A. cordifolia* leaves at doses of 500 mg/kg and 1000 mg/kg body weight was evaluated with two antiulcer testing models (pylorus ligation and HCI/ethanol-induced) in rats. The results indicated that the methanolic extract at dose of 1000 mg/kg body weight prevented ulcer formation and caused a marked reduction in gastric juice secretion [44]. Osafanme et al. investigated a polyherbal formulation of the aqueous extract of *A. cordifolia* (EXR-HF2) on indomethacin, ethanol, and histamine ulcer models, and the result revealed that EXR-HF2 had gastroprotective potential which hindered the creation of ulcer induced in all the three ulcer models [45]. The ethanolic extract of *A. cordifolia* leaves has also demonstrated great potential in the treatment and management of gastric ulcers caused by *H. pylori* [46].

### 3.3. *Blighia sapida* K.D. Koenig

*Blighia sapida* (*B. sapida*) is an evergreen plant that is domesticated in West Africa. It normally grows to about 25 m high. It belongs to the family Sapindaceae and is commonly referred to as Ackee in English. The fruit from ackee is consumed fresh, roasted, or dried. Some natives use the fruit for soup or sauce in some communities in West Africa [47, 48]. The *Blighia sapida* tree is used in folk medicine for the management of hernia, yellow fever, malaria, diabetes, dysentery, internal hemorrhage, bleeding piles, epilepsy, oedema, diarrhea, migraine, yaws, and ulcers [49–51]. Ojo et al. assessed the antioxidant and inhibitory properties of the stem bark, characterized the phytochemicals, and concluded that the ethanolic extract of the stem bark possesses the inhibitory effects of *α*-glucosidase and *α*-amylase in addition to its antioxidative effect [52]. The inhibitory effects of the *B. sapida* stem bark extract on the *α*-amylase and *α*-glucosidase actions may be a result of its phytochemical contents such as polyphenols, tannins, and saponins [52]. The root extract of *B. sapida* has been shown to have a hypoglycaemic effect in normoglycemic rats and ameliorate pancreatic *β*-cell dysfunction [53]. The phytochemical constituents of the stem bark include cardiac glycosides, saponins, anthraquinones, flavonoids, alkaloids, phlobatannins, and terpenes [54].

A study conducted by Oreagba et al. to analyse the antiulcer activity of the hydroethanolic leaf extract of *Blighia sapida* using absolute HCl-ethanol, ethanol, cold-restraint stress, indomethacin, and pylorus ligation ulceration in rats showed that the extract produced dose-relative antiulcer effects [55]. This was evident by the significant protection of the gastric membrane. The hydroethanolic leaf extract also markedly reduced the ulcer index in the cold-restraint stress and pyloric ligation-induced ulcer models compared to cimetidine and omeprazole standard drugs. Also, the levels of the pH, gastric juice volume, free acidity, and acidity determined in the ulcer model induced by pylorus ligation demonstrated that pretreatment with the hydroethanolic leaf extract of *Blighia sapida* safeguarded gastric mucosa by marked reduction of acid secretion and the ulcer index in gastric membrane injury. *B. sapida* may therefore have antisecretory, gastroprotective, and free radical scavenging properties [55].

Adedosu et al. also evaluated the aqueous leaf extract of *Blighia sapida* on ethanol-induced gastric ulcer in male Wistar rats where animals treated with the extract at doses of 400 mg/kg body weight showed the maximum inhibition of the ulcer index. Results from the study indicated that *Blighia sapida* leaves showed ameliorative, antiulcer, and anti-inflammatory activities. These exhibited actions may be due to phytochemical constituents in the plant in addition to antisecretive effects of the aqueous extract of *Blighia sapida* [56].

### 3.4. *Cyperus rotundus* L.

*Cyperus rotundus* (*C. rotundus*), a perennial herb found in the tropical and temperate regions, is from the Cyperaceae family. It is used in folkloric medicine for the management and treatment of malaria, fever, rheumatism, arthritis, pains, and hypertension [57].

The extract of the plant contains some secondary metabolites including tannins, alkaloids, triterpenoids, flavonoids, phenolics, saponins, proteins, essential oils, starch, carbohydrates, and cardiac glycosides [58, 59]. Pharmacological screening confirms that the plant has antiparasitic, anti-inflammatory, antirheumatic, antidiabetic, antidiarrhoeal, antifungal, antispasmodic, neuropharmacological, and aphrodisiac properties [60–62].

*Cyperus rotundus* exhibited gastroprotective potential when tested in Wistar albino rats via the pylorus ligation-induced ulcer model [63]. Also, *C. rotundus* exhibited an antiulcer property when used at a dose of 500 mg/kg against the aspirin-induced ulcer model [62]. Mohammed et al. studied the antiulcer effects of the tuber powder of *C. rotundus* at a dose of 1.25 mg/kg body weight against a histamine-induced gastric ulcer in guinea pigs for 45 minutes and oral administration of 500 mg/kg against the aspirin-induced gastric ulcer for one hour. From the results obtained, a reduction in the ulcer index was observed in both cases which may be attributed to the secondary metabolites of *C. rotundus* [64].

### 3.5. *Azadirachta indica* A. Juss.

*Azadirachta indica* (*A. indica*), commonly referred to as neem, is from the Meliaceae family. It is an evergreen tree originating from South East Asia which also grows in semitropical and tropical areas globally. It is also found in several African countries. *Azadirachta indica* is used in folklore medicine for the treatment of intestinal disorders, skin diseases, constipation, respiratory disorders, malaria [65, 66], fever, asthma, piles, diabetes, urinary tract infections, scabies, and ringworm [66]. The phytochemical constituents found in *A. indica* include alkaloids, saponins, tannins, and flavonoids [67]. The reported pharmacological activities on the leaves of *Azadirachta indica* are comprised of antiulcer, antifungal, anti-inflammatory, antibacterial [68], antiviral, antioxidant, antimutagenic, and antimalarial properties [69]. The antiulcer activity of *Azadirachta indica* has been reported by Chattopadhyay et al. [70], where the aqueous leaf extract was used to prevent gastric ulcers, oxidative damage, acid secretion, and apoptosis in rats. The antiulcer models employed were pylorus ligation, cold stress, ethanol, and indomethacin-induced models. Results from the ulcer model induced by stress showed the *Azadirachta indica* extract to be more efficacious than ranitidine but less efficacious than omeprazole. The extract also showed an effective reduction in mercaptomethylimidazole-induced acid secretion and pylorus ligation and produced an antiulcer function by preventing the secretion of acid through H+-K+-ATPase and by preventing apoptosis and oxidative damage [70]. The alcoholic extract of *Azadirachta indica* leaves investigated in aspirin-induced gastric ulcers in rats exhibited a reduction in ulcers with pretreatment of the *Azadirachta indica* extract producing a substantial gastroprotective impact in rats [71]. However, a reduction in ulceration may be due to the enhancement of the prostaglandin and cyclooxygenase pathway which makes the gastric membrane more impervious to damage. Prevention of blockage of mucosal membrane function and enhancement of cytosolic adenosine triphosphate activity, reduction in proton dissipation from surface epithelial cells, and prevention of sodium transport may all contribute to the observed antiulcer activity [72]. *Azadirachta indica* may also cause enhancements in mucin, surface-active phospholipids, and bicarbonate secretion. It may also prevent injury resulting from free radicals [69]. The antiulcer activity was also evaluated by Bhajoni et al., [10] using the aqueous leaf extract of *A. indica* at doses of 150, 300, and 600 mg/kg body weight in Wistar rats with three antiulcer models: cold-restraint stress, pylorus ligation, and aspirin. Their study demonstrated that the aqueous extract of *A. indica* showed a marked reduction in the ulcer index in a dose-dependent manner and an increase in percentage inhibition in all models employed compared to the control group using ranitidine as a standard drug. This indicates the likely involvement of the prostaglandin pathway in the medium of the function of *A. indica*. There was also a marked decrease in gastric volume and the free and total acidity in comparison with the control group [10]. Prevention of the production of acid in the stomach and the release of histamine could also cause a reduction in ulceration [73]. *A. indica* extracts have again been investigated on gastric secretion and gastroduodenal ulcer. The indomethacin-induced ulcer model was investigated by Raji et al. [74] using the aqueous stem bark extract of *A. indica*, at doses between 100 and 800 mg/kg body weight. Marked reduction in ulcer was seen. Also, oral administration of 250 mg/kg body weight of *A. indica* in combination with cimetidine (0.12 mg/kg body weight) and histamine (1 mg/kg body weight) *in vivo* resulted in effective inhibition of histamine-induced gastric acid secretion. The *A. indica* extract has also been evaluated in Indian patients suffering from gastroduodenal ulcer and acid-related problems. The extract was dispensed at a dosage regimen of 30 mg twice daily for a total of 6 weeks. Results obtained confirm that the bark extract of *A. indica* has therapeutic potential for the management of gastro/duodenal ulcers. The healing process of duodenal ulcers was monitored using endoscopy and barium meal X-ray. The study also revealed that gastric hypersecretion was adequately controlled [75]. The *A. indica* extract was studied in albino rats and dogs using a chronic ulcer model induced by acetic acid. The results revealed nimbidin, a phytochemical constituent of *A. indica*, may enhance ulcer healing in experimental animals [76].

### 3.6. *Calotropis procera* (Aiton) W.T. Aiton

*Calotropis procera* (*C. procera*) from the family Apocynaceae is a perennial shrub originating from Asia. It is widely found in Africa; Brazil; and North, Western, and Central India [77]. The plant has several folkloric medicinal uses including the management of fever, asthma, piles, liver disorders, dyspepsia, cold, diarrhea, leprosy, indigestion, ulcers, eczema, skin disease, intestinal worms, diarrhea, rheumatism, pain, cough, spleen disorders, tumors, elephantiasis, and jaundice [77–80]. The phytochemical constituents of the plant such as flavonoids, polyphenolic compounds, stigmasterol, *β*-sitosterol, alkaloids, saponins, and tannins have been reported [77]. Pharmacological studies have reported antidiarrheal, antimicrobial, anti-inflammatory, acaricidal, anthelmintic, schizonticidal, insecticidal, and anticancer activities [81, 82]. Antiulcer studies involving the investigation of *C. procera* extract against aspirin- and ethanol-induced ulcer models in albino rats at 200 and 400 mg/kg doses showed marked antiulcer activity in the open excised rat stomach when compared to the standard drugs [83].

Another investigation of the leaf extract of *C. procera*, at a dose of 400 mg/kg, produced a cytoprotective effect against ulcer induced by indomethacin in Sprague-Dawley rats [84]. The *C. procera* leaf extract also showed a reduction in gastric volume and acid accumulation [85]. An investigation of the antiulcer activity in the pylorus ligation model in rats has also shown that the *C. procera* leaf extract caused a reduction in gastric volume [86]. Such promising activities can be linked to the phytoconstituents of each extract. These studies suggest that *C. procera* may have natural potency and could be a promising drug or supplement for the management of peptic ulcers [84]. The antiulcer properties of *C. procera* investigated in rats using aspirin-induced ulceration showed an effective reduction in ulceration. Hence, the antiulcer properties of the extract of *C. procera* can be attributed to free radical scavenging property, reduction in acid secretion, enhancement of gastric mucosal barrier, and improvement in cytoprotection. Marked cytoprotection has also been observed in guinea pigs with the extract of *C. procera* in histamine-induced duodenal ulcers [84].

### 3.7. *Hoslundia opposita* Vahl Family

*Hoslundia opposita* (*H. opposita*) from the Labiatae family is a scrambling soft shrub growing up to about 1.2-4.5 m tall with a wide distribution in the southern part of Africa and tropical Africa. *H. opposita* grows well in both wet and dry areas in Ghana. In folkloric medicine, the plant has been used in remedies of diabetes, cold, malaria, sores, skin diseases, fevers, sore throat, herpes, microbial infections, venereal diseases, epilepsy, wounds, inflammation, and gonorrhea [87–90]. The phytochemical constituents found in the plant include alkaloids, tannins, flavonoids, phenols, terpenoids, saponins, and quinones in both aqueous and ethanolic extracts [91, 92]. The pharmacological activities that have been reported on *H. opposita* include antibacterial, antimalarial, anticonvulsant, and antimicrobial activities; it also has antioxidant, CNS depression, hepatoprotective, and insecticidal properties[91–94].

A decoction from the roots and leaves of *H. opposita* is employed orally to manage chronic and deep wounds, stomach ulcer, and dermatitis in some regions of Ghana [95, 96]. The wound healing activity demonstrated by most plants is linked to the additive or synergistic actions of their constituents [95]. A survey done by Agyare et al. [96] showed that traditional healers used *Hoslundia opposita* for healing wounds and stomach ulcers. Relative adhesion of the *H. opposita* aqueous extract against *Helicobacter pylori* was demonstrated in AGS human stomach cells. The result showed strong antiadhesive agents with relative values of 134 ± 14 mg/ml and 48 ± 12 mg/ml were comparable to a positive control (*Abelmoschus esculentus* fruit extract) with values of 9 ± 6 mg/ml, indicating that the aqueous extract of *H. opposita* may be useful for the treatment of stomach ulcer [95]. The antiulcer activities on *H. opposita* are scarce; however, essential oils present in the plant may contribute positive effects in the treatment of stomach ulcer pain [97]. It has been found that flavonoids exhibit antimicrobial activity and consequently may significantly help to reduce or prevent wound infections [98].

### 3.8. *Kigelia africana* (Lam.) Benth

*Kigelia africana* (*K. africana*) is from the Bignoniaceae family and is a perennial tree (sausage tree) growing in the tropics and subtropics of Africa. It is widely spread in the coastal and Savanna Regions of Ghana [99].

The bark, root, and leaf of *K. africana* have been locally used for skin diseases, depression, diabetes, fungal infection, wound, eczema, and bacterial infection [100, 101]. The phytochemical constituents found in *K. africana* include reducing sugars, saponins, flavonoids, alkaloids, phytosterols, coumarin, naphthoquinones, and glycosides [101–104]. The reported pharmacological properties of the plant include anticancer, antimalaria, antibacterial, anti-inflammatory, and antioxidant properties [105, 106]. The antiulcer activity of the methanol extract of *K. africana* against gastric injuries induced by aspirin and histamine was reported by Anvesh et al. [107]. The results from their study showed that, after the induction of ulcer, the highest-dose extract (1000 mg/kg body weight) recorded a marked decline in the ulcer index much better than that of ranitidine. In Wistar albino rats, after the induction of ulcer, 100 mg/kg and 400 mg/kg doses of the *K. africana* leaf extract administered with aspirin prevented ulceration. The antiulcer activity can be due to the release of prostaglandins that protect the gastric mucosa and possibly free radical scavengers [108]. The methanolic leaf extracts of the plant in aspirin-induced and ethanol-induced ulcer models in animals have been evaluated by Hamamalini et al. [109]. The methanol leaf extract decreased the ulcer index, ulcer score, and the number of ulcers in aspirin-induced and ethanol-induced ulcer models in rats at a dose of 200 mg/kg. The study also demonstrated that the presence of phytochemical constituents contributed to the reduction in the ulcer index in rats. The reduction in ulceration showed by the methanolic extract of *K. africana* leaves with the aspirin-induced ulcer model in rats could be due to the inhibition of changes in the permeability of the gastric mucosa, the penetration (diffusion) of gastric acid, and the release of histamine [110]. Improving the synthesis of prostaglandin and activating cyclooxygenase can also contribute to the activity observed [111]. At a dose of 450 mg/kg body weight, the ethanolic leaf extract of *K. africana* exhibited cytoprotection in an ethanol-induced ulcer model. There was also a marked decrease in the ulcer index in albino rats in aspirin-induced ulcers equivalent to that of the standard medication, cimetidine [102]. The antiulcer activity on *K. africana* has again been investigated against oxidative stress on ethanol-induced ulcer models in rats. The aqueous leaf extract of the plant orally administered at various dosages after inducing ulcer with ethanol caused a decrease in the ulcer index in a dose-dependent manner. The study, therefore, concluded that the aqueous leaf extract of *K*. *africana* has antiulcer activity [99].

### 3.9. *Spathodea campanulata* Buch.-Ham. ex DC

*Spathodea campanulata* (*S. campanulata*) is also from the Bignoniaceae family. It is a perennial shrub that grows in the subtropical and tropical areas in Africa. The whole plant (leaves, roots, stems, bark, and flowers) are used in folklore medicine as remedies for painful inflammations, stomach aches, diarrhea, kidney diseases, and urethral inflammations and as a diuretic [112]. The phytochemical constituents present in *S. campanulata* include saponins, tannins, anthraquinones, glycosides, phenols, carbohydrates, flavonoids, sterols, and triterpenoids [113, 114]. Pharmacological studies have revealed that the plant possesses analgesic, anti-inflammatory, antiplasmodial, anti-HIV, and antimalarial properties [114–117].

The antiulcer function *in vivo* of the ethanolic extract of *S. campanulata* was investigated against aspirin-induced gastric ulcer models in rats at doses of 200 mg/kg and 400 mg/kg, with results showing substantial decreases in gastric volume, ulcer index, and free acidity [118]. This may be due to the phytochemical constituents present in the plant. Alcoholic and aqueous stem bark extracts of *Spathodea campanulata* have been reported by Radika as possessing antiulcer activity when tested in pylorus ligation, forced swim stress-induced, and indomethacin antiulcer models in Wistar albino rats [119]. The results demonstrated a markedly reduced ulceration in all three models. An increase in defensive factors like mucin and a decrease in aggressive factors like pepsin and pH were observed with testing involving the alcoholic extract. The presence of phytochemical constituents like flavonoids can avoid lipid peroxidation and prevent the degradation of gastric tissues by reactive oxygen reactive organisms [119].

### 3.10. *Mangifera indica* L.

*Mangifera indica* (*M. indica*) from the Anacardiaceae family is an evergreen tree generally growing in tropical and savanna areas reaching a height of between 10 and 45 m. Ethnomedicinal uses of some parts (leaves, bark, roots, fruits, and flowers) of the plant have been reported in the treatment of disorders including cough, hemorrhages, wounds, diarrhoea, ulcers, anorexia, dyspepsia, chronic dysentery, anemia, piles, kidney stones, tuberculosis, intestinal disorders, blood purification, nasal bleeding, and sunstroke [120, 121]. The reported phytochemical constituents include vitamins, carotenoids, polyphenols, sterols, amino acids, flavonoids, and terpenes [122]. The reported pharmacological properties include antidiarrheal, gastroprotective, antioxidant, antidiabetic, hypolipidemic, anticancer, antiparasitic, antifungal, anti-HIV, antibacterial, and antispasmodic properties [120, 123, 124].

Ethanol and petroleum ether extracts obtained from *Mangifera indica* were investigated *in vivo* using an aspirin-induced ulcer animal model. The results showed an effective reduction in the ulcer index [125], which could be attributed to the release of prostaglandins due to enhanced synthesis through the cyclooxygenase pathway in addition to ensuring bicarbonate and mucus secretion and decreased production of reactive oxygen species. It has been reported that *Mangifera indica* provides gastroprotection against gastric ulcers due to its cytoprotective, antioxidative, and antisecretory effects [126]. The ulcer index was substantially reduced compared to the standard drug ranitidine when the leaf extract was examined in rats with the pylorus ligation ulcer model. It can be inferred that the gastroprotective effect for the tested *Mangifera indica* extract*s* may be due to the synergistic effect of all secondary metabolites available in the leaves of the plant [127]. The ethanolic extract's antiulcer function obtained from the *M. indica* kernel evaluated by Prabhu using the pylorus ligation antiulcer model has demonstrated a marked reduction in ulceration [128]. Antiulcer tests involving the decoction of *M. indica* showed a marked reduction in gastric injury from 89.0 ± 6.71 (control group) to 9.25 ± 2.75, 4.50 ± 3.30, and 0, respectively, in the ethanol-induced ulcer model, when decoction was administered orally at doses of 250, 500, and 1000 mg/kg in rats. Also, pretreatment of mice with the decoction by stress-induced and HCl/ethanol gastric ulcer models exhibited a significant reduction in the ulcer index [129]. This could be a result of the prevention of ethanol penetration through the gastric membrane, reduction in acid secretion, and prevention of the generation of reactive oxygen species. Enhanced bicarbonates and mucus production may also lead to the antiulcer effects of *M. indica*.

### 3.11. *Paullinia pinnata* Linn.

*Paullinia pinnata* (*P. pinnata*) from the family Sapindaceae is a climbing plant with woody stiff stems which normally grows in subtropical and tropical areas of Africa. Traditionally, all parts of the plant (leaves, roots, stems, and flowers) are used as a medicine for the treatment of typhoid fever, erectile dysfunction, malaria, dysentery, syphilis, eye disorders, abdominal pains, diarrhoea, wounds, gonorrhea, and waist pains [130–132]. The reported phytochemical constituents in *Paullinia pinnata* include flavonoids, alkaloids, cardiac glycosides, saponins, tannins, carbohydrates, sterols, triterpenoids, and steroidal glycosides [133–135]. The reported pharmacological studies include antiparasitic, antispasmodic, antimalarial, anti-inflammatory, and antimicrobial activities [132, 135, 136]. Antiulcer activity on *Paullinia pinnata* is scarce; however, the plant has been reported to treat stomach ache, wounds, and sores. For instance, *P. pinnata extract* ointment was investigated in *in vitro* and *in vivo* models of male Sprague-Dawley rats against incision and excision wound models in rats where *P. pinnata* ointment was administered. The results observed was a decrease in the epithelialization period by 47.6% with a marked decrease in the scar area by 48.3% compared to control. The significant wound healing action comparable to nitrofurazone (conventional wound healing remedy) proved that the methanolic extract of *P. pinnata* ointment supports wound healing [137]. Hence, the antiulcer activity of *P. pinnata* could be attributed to its free radical scavenging and antioxidant properties as well as phytochemicals such as alkaloids and tannins [131, 138].

### 3.12. *Strophanthus hispidus* DC

*Strophanthus hispidus* (*S. hispidus*) from the family Apocynaceae is a perennial shrub that generally grows in Africa. Several studies suggest that the entire plant (stem bark, leaves, and roots) has numerous therapeutic applications including the treatment of skin diseases, gonorrhea, dysentery, leprosy, diabetes, oedema, malaria, ulcers, rheumatism, and urine retention [139, 140]. The plant contains tannins, glycosides, saponins, flavonoids, alkaloids, resins, and rhamnose sugar [141]. The pharmacological properties investigated on *Strophanthus hispidus* include hypoglycemic, anti-inflammatory, antioxidant, and antimicrobial activities [142–144]. Ishola et al. reported antiulcer activity on the ethanolic extract obtained from the root of *S. hispidus* investigated in albino rats in three ulcer models, namely, ethanol, HCl, and pylorus ligation [145]. Results from the ethanol-induced ulcer model indicated a marked reduction in ulceration compared to misoprostol, a standard drug. *S. hispidus* probably exhibits cytoprotective properties as a result of the release of free radical scavengers. Pharmacological reports show that the plant possesses antioxidant and antisecretory activities. Results from the HCl-induced ulcer model indicated a marked reduction in ulceration comparable to cimetidine, a standard drug. Moreover, in the pyloric ligation-induced ulcer model, *S. hispidus* extract showed an effective reduction in gastric ulcers compared to omeprazole. Reduced ligation of gastric acid and its accumulation in the stomach of the albino rats were also observed. This explains that the *S. hispidus* extract has significant antiulcer properties in various animal models [145].

Investigations conducted in the aspirin-induced ulcer model using an aqueous antiulcer drug formulated with *S. hispidus* as a constituent in albino rats demonstrated that the formulated drug markedly reduced ulceration compared to omeprazole, a standard drug. This could be because of the effective scavenging of free radicals' production due to the adequate antioxidant property of *S. hispisdus*. Also, reduced acid secretion and enhanced prostaglandin biosynthesis through the cyclooxygenase pathway ensured the bicarbonate and mucus secretion and decreased production of reactive oxygen species [146].

### 3.13. *Zingiber officinale* Rosc.

*Zingiber officinale* (ginger) is a perennial horizontal rhizome from the family Zingiberaceae. It grows up to 1.5 to 3 feet tall. Ginger is native to India but now grows well in several African countries including Ghana. *Zingiber officinale* is used in traditional medicine as remedies for pain, rheumatism, diarrhoea, bronchitis, nausea, influenza, vomiting, asthma, hemorrhoids, bloated stomach, anemic conditions, chronic osteoarthritis, and diabetes [147–149]. Phytochemical constituents found in *Zingiber officinale* include alkaloids, steroids, terpenoids, flavonoids, fats and oil, resins, and carbohydrates [150].

*Zingiber officinale* has been found to possess antimicrobial, antibacterial, antidiabetic, antiemetic, antifungal, anthelmintic, anti-inflammatory, antithrombotic, antiviral, antitumor, antitussive, antiulcer, and antioxidant properties [151, 152].

The antiulcer activity on the ethanolic extract of *Zingiber officinale* was investigated in adult Wistar rats by the use of the indomethacin-induced ulcer model. The result showed that the ethanolic extract of ginger at doses of 100, 200, and 400 mg/kg exhibited a decrease in gastric wounds in rats with an ulcer inhibition of 71.56%, 48.53%, and 53.40%, respectively, when compared to 100 mg/kg body weight of the standard drug, ranitidine, which has an ulcer inhibition of 78.40%. The reduction in the gastric ulcer induced by indomethacin in the Wistar rats could be attributed to the increased production of prostaglandins in the cyclooxygenase pathway, ensuring bicarbonate and mucus secretion, decreased reactive oxygen species production, and inhibition of pepsin and acid secretion [153]. Zaman et al. also reported antiulcer actions in indomethacin-induced gastric lesions in rats with the aqueous extract of *Zingiber officinale* [154]. The result exhibited a marked decrease in gastric ulcer at dosage quantities of 200 mg kg^−1^ and 400 mg kg^−1^ when compared with omeprazole at a dosage quantity of 10 mg/kg. Also, relative gastric ulcer inhibition of 40.91% and 57.58% compared to that of omeprazole which was 65.91% was obtained. This confirms that the aqueous *Zingiber officinale* extract exhibits antiulcer activity in the indomethacin-induced ulcer model investigated [154]. The aqueous *Zingiber officinale* extract was again evaluated in albino rats using the ulcer model by indomethacin. The result indicated that administration of the aqueous extract of *Zingiber officinale* orally at a regimen of 0.8 mg/kg body weight for a total of 14 days demonstrated a significant reduction in ulcer index comparable to omeprazole, a standard drug [155]. It could be due to the enhancement of the mucosal barrier and prevention of excessive acid secretion, increase in endogenous prostaglandin biosynthesis, and enhancement in mucus and bicarbonate production [156].

In another investigation, the antiulcer activity of the steamed extract of ginger was investigated in rats using the ethanol and HCl-induced gastric ulcer models. The steamed extract of *Zingiber officinale* was administered orally for 14 days to rats before administration of ethanol and HCI to induce gastric injury in the experimental animal. The result showed that the pretreatment prevented gastric damage and enhanced the production of mucosal total nitrite/nitrate in gastric tissues. The findings concluded that steamed ginger extract may exhibit antiulcer activity in a rat model by reducing acute gastric ulcers. The preventive mechanism probably comes as a result of an increase in mucosal prostaglandin E2 synthesis and gastric antioxidation capability [157].

## 4. Discussion

Generally, peptic ulcer disease affects 5-10% of people globally [1]. Currently, due to the changes in our diet and because of emotional and social factors, there is a rise in this disease. A lot of orthodox medications have been manufactured for the management and or treatment of this disorder. Most of these medications are expensive and have side effects when taken. Herbal medications have been used in treating several diseases from times past before the discovery of most orthodox medicines. As a result, herbal medicines have been considered as having healthier cultural acceptability and having less harmful and less antagonistic effects [4]. Consequently, herbal medicines have become very attractive sources of new drugs and have demonstrated promising results in the treatment of various diseases including peptic ulcers. Several plants have been known to have folkloric use in the treatment of peptic ulcer disease, but not much is known about them.

In this review, thirteen plants, namely, *Chromolaena odorata*, *Blighia sapida*, *Cyperus rotundus*, *Magnifera indica*, *Azadirachta indica*, *Alchornea cordifolia*, *Calotropis procera*, *Hoslundia opposita*, *Kigelia africana*, *Spathodea campanulata*, *Zingiber officinale*, *and Paullinia pinnata* were assessed for their phytochemical constituents and pharmacological studies.

From this study, two of the plants under review, namely, *Kigelia africana* and *Spathodea campanulata* belong to the family Bignoniaceae. Further studies could be done to assess the antiulcer activity of other plants belonging to this plant family since these two might not be the only ones. Also, the two plants have some similar chemical constituents including flavonoids, saponins, sterols, glycosides, and quinones. Flavonoids and saponins, for instance, have been proven to have antiulcer activities [158]. Antiulcer studies could be carried out for plants that possess these two chemical constituents to ascertain the possibility of this suspected effect.

As shown in Table 1, several phytochemical constituents have been isolated from the enlisted plants but these are not exhaustive. Additional phytochemical analysis could be performed to source more of these constituents since plants are known to be rich in several components.

All the plants under review except *Paullinia pinnata* have undergone some form of pharmacological studies to confirm their use in peptic ulcer disease (Table 2). For *Hoslundia opposita*, a study has shown its effect on *Helicobacter pylori* but not on any animal ulcer model. Furthermore, among the listed plants in this review, *Azadirachta indica* is the only plant that has undergone patient studies as well as pharmacological animal ulcer model studies. Besides, the antiulcer activity in *C. odorata* has been verified; however, results from a toxicity analysis carried out on a rat model suggested that unregulated use of this plant extract may have negative effects on kidney function and intestinal histology [32]. Hence, doses for formulations must be carefully selected with accompanying toxicological evaluations of finished products containing *C. odorata* extracts. Further studies could be done to fill the above-listed gaps, namely, to employ other animal ulcer models which were not used for each plant to assess its activity, to employ human studies for all plants, and lastly to ascertain the ulcer preventive or treatment effect of *Paullinia pinnata.*

## 5. Conclusion

The review has successfully established certain potential plants available in Ghana which, in addition to having other pharmacological properties, have substantial antiulcer activity in animal models and certain clinical settings. These medicinal plants could be used individually or in combination for the formulation of herbal medicines for remedies of peptic ulcer disease in Ghana.

## Figures and Tables

**Table 1:** Reported phytochemical constituents isolated from the list of ethnomedicinal plants with folkloric use for peptic ulcer disease.

Plant name	Family	Part used	Phytochemical constituents	References
(1) *Chromolaena odorata* (*C. odorata*)	Asteraceae	Leaves	Alkaloids, flavonoids, tannins, steroids, terpenoids, and cardiac glycosides	[24–27].
(2) *Alchornea cordifolia* (*A. cordifolia*)	Euphorbiaceae	Leaves	Tannins, terpenoids, glycosides, alkaloids, saponins, and flavonoids	[43].
(3) *Blighia sapida* (*B. sapida*)	Sapindaceae	Leaves/stem bark	Cardiac glycosides, saponins, anthraquinones, flavonoids, alkaloids, phlobatannins, and terpenes	[54]
(4) *Cyperus rotundus* (*C. rotundus*)	Cyperaceae	Whole plant	Tannins, alkaloids, triterpenoids, flavonoids, phenolics, saponins, proteins, essential oils, starch, carbohydrates, and cardiac glycosides	[58, 59]
(5) *Azadirachta indica* (*A. indica*)	Meliaceae	Leaves	Alkaloids, saponins, tannins, and flavonoids	[67]
(6) *Calotropis procera* (*C. procera*)	Apocynaceae	Leaves	Flavonoids, polyphenolic compounds, stigmasterol, *β*-sitosterol, alkaloids, saponins, and tannins	[77]
(7) *Hoslundia opposita ***(***H. opposita*)	Labiatae	Leaves and roots	Alkaloids, tannins, flavonoids, phenols, terpenoids, saponins, quinones in both aqueous and ethanolic extracts	[91, 92]
(8) *Kigelia africana* (*K. africana*)	Bignoniaceae	Leaves	Reducing sugars, saponins, flavonoids, alkaloids, phytosterols, coumarin, naphthoquinones, and glycosides	[101–104]
(9) *Spathodea campanulata* (*S. campanulata*)	Bignoniaceae	Stem bark	Saponins, tannins, anthraquinone glycosides, phenols, carbohydrates, flavonoids, sterols, and triterpenoids	[113, 114]
(10) *Mangifera indica* (*M. indica*)	*Anacardiaceae*	Leaves	Vitamins, carotenoids, polyphenols, sterols, amino acids, flavonoids, and terpenes	[122]
(11) *Paullinia pinnata* (*P. pinnata*)	Sapindaceae	All parts	Flavonoids, alkaloids, cardiac glycosides, saponins, tannins, carbohydrates, sterols, triterpenoids, and steroidal glycosides	[133–135]
(12) *Strophanthus hispidus* (*S. hispidus*)	Apocynaceae	Roots	Tannins, glycosides, saponins, flavonoids, alkaloids, resin, and rhamnose sugar	[141]
(13) *Zingiber officinale* (ginger)	Zingiberaceae	Rhizome	Alkaloids, steroids, terpenoids, flavonoids, fats and oil, resins, and carbohydrates	[150]

**Table 2:** Reported pharmacological peptic ulcer studies (ulcer models and clinical studies) done on the list of ethnomedicinal plants with folkloric use for peptic ulcer disease.

Plant Name	Family	Part used	Pharmacological studies	References
(1) *Chromolaena odorata* (*C. odorata*)	Asteraceae	Leaves	Ethanol-induced ulcer model	[30, 31]
(2) *Alchornea cordifolia* (*A. cordifolia*)	Euphorbiaceae	Leaves	Pylorus ligation, HCI/ethanol-induced, indomethacin, ethanol, and histamine ulcer models	[44–46]
(3) *Blighia sapida* (*B. sapida*)	Sapindaceae	Leaves	HCl-ethanol, ethanol, cold-restraint stress, indomethacin, and pylorus ligation ulcer models	[55]
(4) *Cyperus rotundus* (*C. rotundus*)	Cyperaceae	Whole plant	Pylorus ligation, aspirin, and histamine-induced gastric ulcer models	[62–64]
(5) *Azadirachta indica* (*A. indica*)	Meliaceae	Leaves	Pylorus ligation, cold-restraint stress, ethanol, indomethacin, aspirin, and acetic acid-induced ulcer models In patients, the bark extract of *A. indica* has therapeutic potential for the management of gastro/duodenal ulcers	[10, 70, 71, 74, 75]
(6) *Calotropis procera* (*C. procera*)	Apocynaceae	Leaves	Aspirin, ethanol, indomethacin, pylorus ligation, and aspirin-induced ulcer models	[83, 84, 86]
(7) *Hoslundia opposita* (*H. opposita*)	Labiatae	Leaves and roots	*H. opposita* aqueous extract against *Helicobacter pylori* was demonstrated in AGS human stomach cells	[95]
(8) *Kigelia africana* (*K. africana*)	Bignoniaceae	Leaves	Aspirin, histamine, and ethanol-induced ulcer models	[102, 107–110]
(9) *Spathodea campanulata* (*S. campanulata*)	Bignoniaceae	Stem bark	Aspirin, pylorus ligation, forced swim stress, and indomethacin-induced ulcer models	[118, 119]
(10) *Mangifera indica* (*M. indica*)	*Anacardiaceae*	Leaves	Aspirin, pylorus ligation, ethanol, stress, and HCl/ethanol ulcer-induced models	[125, 127–129]
(11) *Paullinia pinnata* (*P. pinnata*)	sapindaceae	All parts		
(12) *Strophanthus hispidus* (*S. hispidus*)	Apocynaceae	Roots	Ethanol, HCl, pylorus ligation, and aspirin-induced ulcer models	[145, 146]
(13) *Zingiber officinale* (ginger)	Zingiberaceae	Rhizome	Indomethacin-induced ulcer models	[153, 154]
